# Altered local spontaneous activity in frontal lobe epilepsy: a resting‐state functional magnetic resonance imaging study

**DOI:** 10.1002/brb3.555

**Published:** 2016-09-21

**Authors:** Li Dong, Hechun Li, Zhongqiong He, Sisi Jiang, Benjamin Klugah‐Brown, Lin Chen, Pu Wang, Song Tan, Cheng Luo, Dezhong Yao

**Affiliations:** ^1^ Key Laboratory for NeuroInformation of Ministry of EducationCenter for Information in Medicine, High‐Field Magnetic Resonance Brain Imaging Key Laboratory of Sichuan ProvinceSchool of Life Science and TechnologyUniversity of Electronic Science and Technology of ChinaChengduChina

**Keywords:** FOur‐dimensional (spatiotemporal) Consistency of local neural Activities, frontal lobe epilepsy, functional magnetic resonance imaging, resting‐state

## Abstract

**Purpose:**

The purpose of this study was to investigate the local spatiotemporal consistency of spontaneous brain activity in patients with frontal lobe epilepsy (FLE).

**Method:**

Eyes closed resting‐state functional magnetic resonance imaging (fMRI) data were collected from 19 FLE patients and 19 age‐ and gender‐matched healthy controls. A novel measure, named FOur‐dimensional (spatiotemporal) Consistency of local neural Activities (FOCA) was used to assess the spatiotemporal consistency of local spontaneous activity (emphasizing both local temporal homogeneity and regional stability of brain activity states). Then, two‐sample *t* test was performed to detect the FOCA differences between two groups. Partial correlations between the FOCA values and durations of epilepsy were further analyzed.

**Key Findings:**

Compared with controls, FLE patients demonstrated increased FOCA in distant brain regions including the frontal and parietal cortices, as well as the basal ganglia. The decreased FOCA was located in the temporal cortex, posterior default model regions, and cerebellum. In addition, the FOCA measure was linked to the duration of epilepsy in basal ganglia.

**Significance:**

Our study suggested that alterations of local spontaneous activity in frontoparietal cortex and basal ganglia was associated with the pathophysiology of FLE; and the abnormality in frontal and default model regions might account for the potential cognitive impairment in FLE. We also presumed that the FOCA measure had potential to provide important insights into understanding epilepsy such as FLE.

## 1 Introduction

As the second most common type of localization‐related epilepsies, frontal lobe epilepsy (FLE) accounts for 20%–30% of all partial epilepsies (Manford, Hart, Sander, & Shorvon, 1992), and it will impact on a broad range of cognitive domains in FLE patients (Braakman et al., 2011, 2012; Exner et al., 2002). There are various origins of frontal lobe seizures including perirolandic, supplementary sensorimotor area, dorsolateral frontal, orbitofrontal, anterior frontopolar, opercular, and cingulate types (Bagla & Skidmore, 2011). However, so far, impacts of FLE on brain functions are not yet fully understood. A wide cognitive dysfunctions and behavioral disturbances in FLE patients, ranging from impairment of executive to the problem of social withdrawal, have been found in various studies (Braakman et al., 2011, 2012; Exner et al., 2002). In the previous task (Braakman et al., 2013) and resting‐state (Cao et al., 2014; Luo, An, Yao, & Gotman, 2014; Widjaja, Zamyadi, Raybaud, Snead, & Smith, 2013) functional magnetic resonance imaging (fMRI) studies, decreased functional connections, which were related with frontal lobes, were reported in FLE patients. Because frontal lobes play crucial roles in a large range of functional domains (Cummings & Miller, 2007), functional abnormalities related with frontal lobes may account for the cognitive dysfunctions in FLE patients (Braakman et al., 2011; Exner et al., 2002). Therefore, while previous resting‐state fMRI studies of FLE focused on the single resting‐state network such as motor network (Woodward, Gaxiola‐Valdez, Goodyear, & Federico, 2014), connectivity pattern of epileptic network (Luo et al., 2014), or relationships between resting‐state networks (Cao et al., 2014; Widjaja et al., 2013), investigation of the local spontaneous activity using FOCA measure may provide important information that will help to understand FLE.

Resting‐state fMRI, as well as its derived functional measures, has been used as a powerful tool for studying spontaneous brain activity and brain functions (Biswal, Yetkin, Haughton, & Hyde, 1995; Fox & Raichle, 2007). For example, low‐frequency resting‐state patterns such as visual or sensory motor cortices were acquired using probabilistic independent component analysis in the FSL software (Beckmann, DeLuca, Devlin, & Smith, 2005; Smith et al., 2004). Unlike task fMRI which only focuses on a single functional system at a time, resting‐state fMRI can provide spontaneous activity information that will be valuable for studying mechanisms of functional alterations in neuropsychological diseases such as epilepsy (Jiang et al., 2016; Luo et al., 2012; Yang et al., 2014), schizophrenia (Chen et al., 2015), and Alzheimer's disease (Wu et al., 2011). Using resting‐state fMRI, it has been suggested that the local functional homogeneity of spontaneous activity has neurobiological relevance which may be determined by anatomical, developmental, and neurocognitive factors (Jiang et al., 2015). Currently, a novel measure, named FOur‐dimensional (spatiotemporal) Consistency of local neural Activities (FOCA), has been proposed to investigate local spontaneous activity based on integrating temporal and spatial information using fMRI (Dong et al., 2015). The FOCA analysis method has several advantages. First, since FOCA measure integrates the temporal and spatial information in a local region (even it may be too strict resulting in some information cannot be detected), it is more flexible to characterize the local spontaneous activity. The FOCA measure emphasizes both temporal homogeneity of local adjacent voxels using temporal correlation, and regional stability of brain activity states between neighboring time points, which may be considered to reflect local functional states. The larger the FOCA value (value from 0 to 1) is, the higher the consistency of local spontaneous activity. Second, it is a data‐driven method without prior knowledge and practical choices of the key parameter settings, thus, it may be suitable for studying a neuropsychological disease without being fully understood. Third, FOCA measure also has a good reproducibility and reliability (Dong et al., 2015). Taken together, the FOCA method perhaps has potential to quantify and uncover the functional changes of local spontaneous activity in a neuropsychological disease such as epilepsy.

The purpose of this study was to investigate local spatiotemporal consistency of spontaneous activity in FLE patients using resting‐state fMRI. Considering epileptic seizures may reflect abnormal neuronal synchronization (Jiruska et al., 2013), FOCA may uncover the impacts of FLE on temporal synchronization and functional states of local brain regions. The FOCA measure was conducted on resting‐state fMRI data of FLE patients and controls. Then, two‐sample *t* test was used to compare the FOCA maps of patients and controls. In addition, the relationships between FOCA alterations and duration of epilepsy in FLE patients were also investigated.

## 2 Materials and Methods

### 2.1 Subjects

A total of 19 FLE patients (9 females/10 males; mean age = 24.2 years; standard deviation = 9.5 years; age range = 13–51 years) who participated in the resting‐state fMRI experiment were recruited from the Center for Information in Medicine, University of Electronic Science and Technology of China (UESTC). All patients were diagnosed by the neurologists (P.W. and S.T.) based on the clinical information consistent with the International League Against Epilepsy (ILAE) guidelines (Engel & International League Against Epilepsy (ILAE), 2001). Routine examinations of CT and MRI scanning showed no structural abnormalities in FLE patients. The detailed demographic information and the clinical characteristics of FLE patients are summarized in Table 1. Age‐ and gender‐matched, healthy subjects were also recruited as controls (a total of 19 controls; 5 females/14 males; mean age = 20.9 years; standard deviation = 8.9 years; age range = 11–41 years). Written consent forms were obtained from each of patients and controls, and the study protocol was approved by the local Ethics Committee of UESTC.

**Table 1:** Demographic and clinical information of frontal lobe epilepsy patients

Patient number	Gender	Age	Age of onset	Interictal EEG	Family history with epilepsy	Antiepileptic drugs
1	M	24	16	R	Brother	CBZ/PIR
2	F	22	12	B	Sister	TPM
3	M	20	10	B	Sister	VPA
4	F	23	16	B	–	LTG
5	F	15	11	B	Brother	a
6	M	23	13	B	–	VPM/PIR
7	F	14	3	B	Sister	CBZ/GAS
8	M	18	7	B	–	TPM/OXC
9	F	32	18	B	–	VPA
10	F	13	10	R	Brother	OXC/PIR
11	M	21	12	L	–	CBZ/TCM
12	F	16	7	R	–	LEV
13	M	25	8	R	–	CBZ
14	M	42	38	L	–	TPM/OXC/TCM
15	F	28	18	B	–	VPM/OXC
16	M	14	11	L	–	OXC/TCM
17	F	31	3	R	Sister	VPA/TPM
18	M	51	51	L	–	b
19	M	27	10	R	–	VPM/PIR

VPA, valproic acid; LTG, lamotrigine; VPM, valpromide; CBZ, carbamazepine; PIR, piracetam; TPM, topiramate; OXC, oxcarbazepine; GAS, gastrodin; LEV, levetiracetam; TCM, traditional Chinese medicine.

a Has no medication for about 2 months.

b Drug naive; M, male; F, female; L, left; R, right; B, bilateral.

### 2.2 MRI acquisition

All MRI data were collected using a MRI scanner (3.0T, Discovery MR750, GE, USA) in the Center for Information in Medicine of UESTC. T1‐weighted anatomical images were collected using a three‐dimensional fast spoiled gradient echo (3D FSPGR) sequence, and the scan parameters were as follows: slices = 152; TR/TE = 6.008 ms/1.984 ms; field of view = 256 × 256 mm^2^; flip angle = 9°; matrix size = 256 × 256 and slice thickness = 1 mm (no gap). The functional images were collected using a gradient‐echo echo‐planar imaging sequence. The scan parameters were as follows: slices = 35; TR/TE = 2,000 ms/30 ms; field of view = 240 × 240 mm^2^; flip angle = 90°; matrix size = 64 × 64 and thickness = 4 mm. A total of 255 volumes were obtained over a 510 s period. During resting‐state fMRI scanning, all subjects were explicitly instructed to close their eyes and relax without falling asleep.

### 2.3 Data preprocessing

For functional images, the first five volumes were discarded to remove the T1 saturation effects. Then, the resting‐state fMRI preprocessing comprised slice time correction, realignment, and spatial normalization (3 × 3 × 3 mm^3^). The analysis of preprocessing was conducted using the SPM8 software (RRID:SCR_007037, http://www.fil.ion.ucl.ac.uk/spm/software/spm8/). The head motion of each subject was assessed using the following formula:

(1) $$\begin{array}{cc} \text{head motion}\;=\; & \frac{1}{N-1}\sum_{i=2}^{N}\\ & \sqrt{{\left|\Delta d_{x_{i}^{1}}\right|}^{2}+{\left|\Delta d_{y_{i}^{1}}\right|}^{2}+{\left|\Delta d_{z_{i}^{1}}\right|}^{2}+{\left|\Delta d_{x_{i}^{2}}\right|}^{2}+{\left|\Delta d_{y_{i}^{2}}\right|}^{2}+{\left|\Delta d_{z_{i}^{2}}\right|}^{2}}, \end{array}$$

where *N* is the number of the fMRI time points; $x_{i}^{1}/x_{i}^{2}$, $y_{i}^{1}/y_{i}^{2}$ and $z_{i}^{1}/z_{i}^{2}$ are translations/rotations at the *i*th time point in the *x*,* y,* and *z* directions, respectively; $\Delta d_{x_{i}^{1}}=x_{i}^{1}-x_{i-1}^{1}$, and similar for others. All patients and controls were translation <1 mm and rotation <1°. Finally, the unsmoothed resting‐state fMRI data were further preprocessed by regressing out linear trend signal, six head motion parameters, individual mean white matter, and cerebrospinal fluid signals.

### 2.4 FOCA analysis

A neuroscience information toolbox (NIT, v1.1, RRID:SCR_014501 http://www.neuro.uestc.edu.cn/NIT.html) was used to calculate the FOCA map of each subject. Briefly, for each voxel, FOCA value was calculated as the product of mean temporal correlation (across cross‐correlation coefficients of adjacent 27 voxels) and mean spatial correlation of a local region (across correlations between local spatial distributions in the neighboring time points). More details of FOCA can be seen in the previous paper (Dong et al., 2015). Unsmoothed versions of FOCA maps were normalized by dividing it by the mean value of the whole brain and spatially smoothed (8‐mm full‐width at half maximum). Then, two‐sample *t* tests were performed to study the difference of normalized FOCA maps between FLE patients and controls (*p* < .05, two‐tailed), while adding age, gender, and head motion as covariables. The results were corrected for multiple comparisons using AlphaSim correction which was based on Monte Carlo simulation algorithm (*p* < .05; Ward, 2000).

### 2.5 Relationships between FLE duration and FOCA

To detect the underlying relationships between normalized FOCA values and duration of epilepsy, partial correlation analysis was conducted in FLE patients, while controlling for age, gender, and head motion. The normalized FOCA values were extracted from the brain regions of significant differences between patients and controls.

## 3 Results

**Figure 1:**
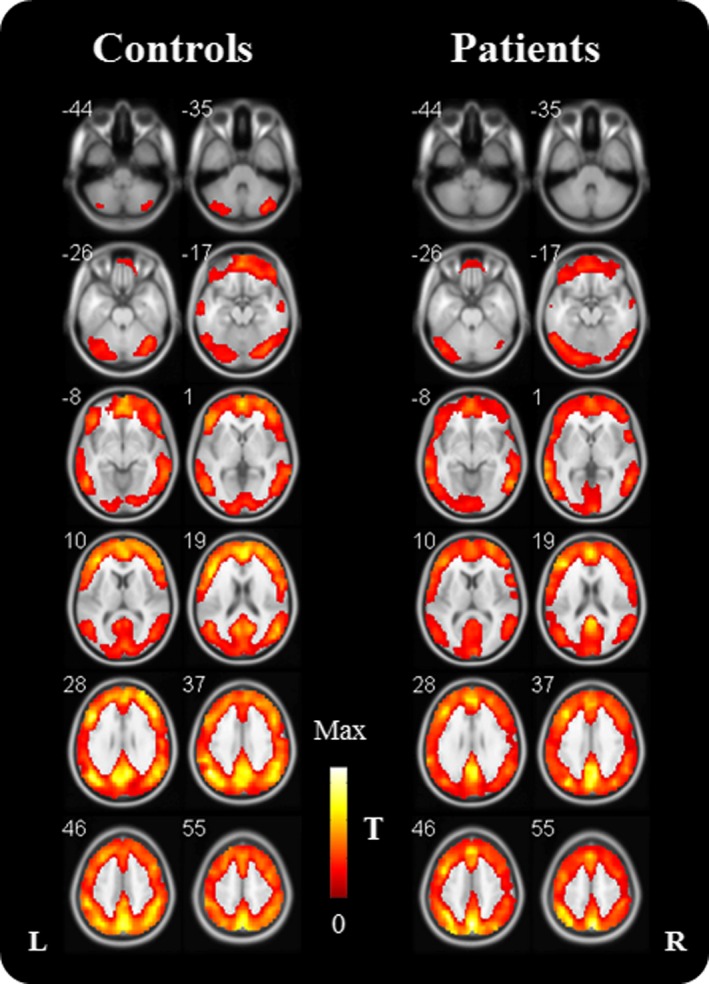
T‐maps of normalized FOur‐dimensional (spatiotemporal) Consistency of local neural Activities (FOCA) in controls and frontal lobe epilepsy patients (One sample *t* test, *p* < .05, false discovery rate corrected). The T‐maps demonstrated brain regions in which FOCA values were significantly greater than the mean FOCA values of whole brain. *T*,* T*‐values; L, left; R, right

As illustrated in Fig. 1, one sample *t* test was conducted on each group to demonstrate the significant brain regions in which FOCA values were greater than the mean FOCA values of the whole brain (*p* < .05, false discovery rate corrected). For both groups, those regions mainly included the bilateral cerebellum, the middle temporal cortex (Brodmann Area BA21/37), the calcarine gyrus (BA17), the lingual gyrus (BA17), the frontal gyrus (BA9/10/45/46), the anterior cingulate (BA32), the precuneus (BA7), and angular gyrus (BA39/BA40).

**Figure 2:**
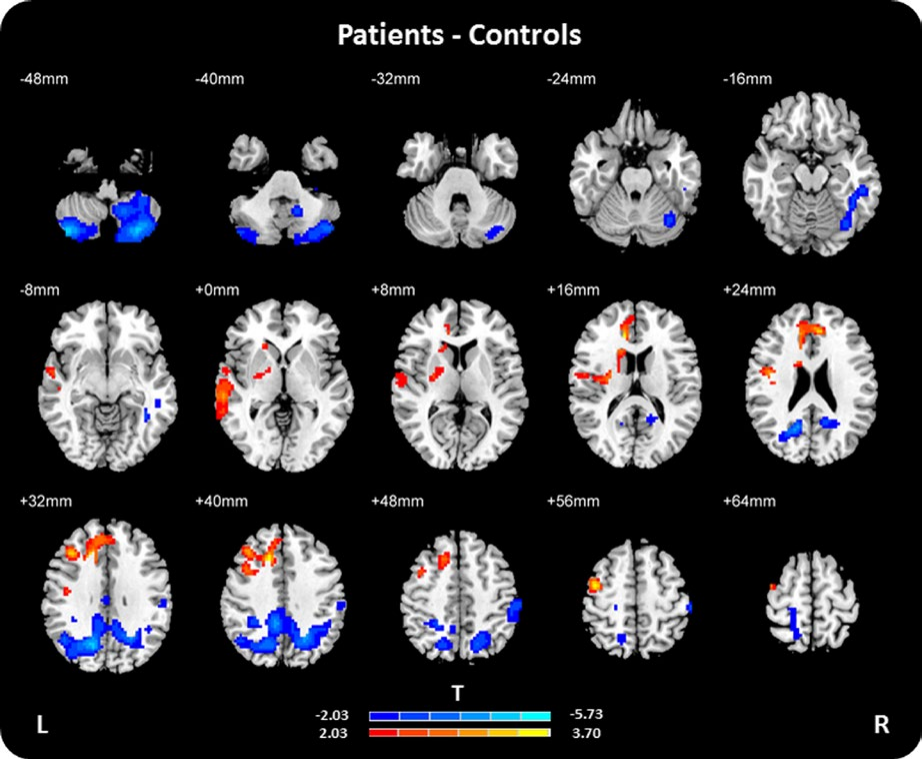
The differences of normalized FOur‐dimensional (spatiotemporal) Consistency of local neural Activities maps between frontal lobe epilepsy patients and controls (two‐sample *t* test, *p* < .05, AlphaSim corrected), while adding age, gender and head motion as covariables. T‐values were showed with hot color for positive values (patients > controls) and cool colors for negative values (patients < controls). L, left; R, right; *T*,* T*‐values

Significant differences between normalized FOCA maps of FLE patients and controls (*p* < .05, AlphaSim corrected) were illustrated in Fig. 2 and Table 2. Compared with controls, increased normalized FOCA values in FLE patients were mainly found in the left precentral gyrus (BA9), the left middle frontal gyrus (BA8/9), the bilateral anterior cingulate (BA32), the left sublobar (BA13), the left middle temporal gyrus (BA21), the left caudate, and putamen. The decreased normalized FOCA values in FLE patients were mainly located in the right temporal lobe (BA37), the bilateral cerebellum posterior lobe, and precuneus (BA7/31).

**Table 2:** Results of two‐sample *t* test between normalized FOur‐dimensional (spatiotemporal) Consistency of local neural Activities maps of frontal lobe epilepsy patients and controls (*p* < .05, AlphaSim corrected), while adding age, gender, and head motion as covariables

	MNI coordinates				Lobe	Brodmann area	*T*‐value	Voxels
	*x*	*y*	*z*	L/R				
Patients > controls	−42	−6	57	L	Precentral gyrus	BA9	3.7	675
	−36	27	36	L	Middle frontal gyrus	BA8/9	3.5	
	6	39	24	R	Anterior cingulate	BA32	3.22	
	−12	33	18	L	Anterior cingulate	BA32	3.45	
	−15	12	21	L	Caudate		3.58	528
	−39	−9	21	L	Sublobar	BA13	3.57	
	−63	−30	0	L	Middle temporal gyrus	BA21	2.95	
	−24	−3	12	L	Putamen		2.52	
Patients < controls	−39	−75	−54	L	Cerebellum posterior lobe		−5.73	581
	30	−75	−51	R	Cerebellum posterior lobe		−4.86	1,471
	42	−45	−12	R	Temporal lobe	BA37	−3.21	
	−18	−66	30	L	Precuneus	BA7/31	−4.82	1,793
	24	−57	39	R	Superior parietal lobule	BA7/31	−4.3	

**Figure 3:**
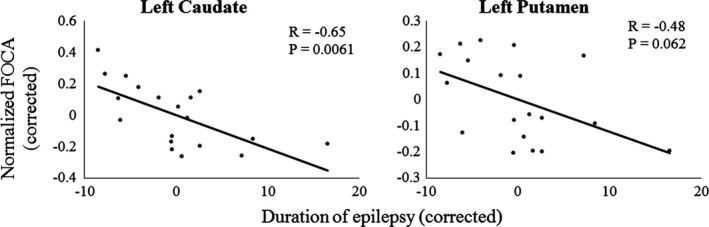
Partial correlations between normalized FOur‐dimensional (spatiotemporal) Consistency of local neural Activities (FOCA) values and the duration of epilepsy in frontal lobe epilepsy patients, while controlling for age, gender and head motion. The normalized FOCA and duration values were corrected by regressing out the age, gender and head motion. *R*, partial correlation coefficient; *p*,* p*‐value

The normalized FOCA values in the brain regions of significant differences were partially correlated with the duration of epilepsy in FLE patients, while controlling for age, gender, and head motion. A significant partial correlation was found in the left caudate (*R* = −.65, *p* = .0061), as well as a marginal significance in the left putamen (*R* = −.48, *p* = .062; Fig. 3).

## 4 Discussion

In this study, we originally used the FOCA metric on resting‐state fMRI to spatiotemporally investigate the alterations of regional spontaneous activity in FLE patients. Compared with controls, FLE patients showed significantly increased FOCA values in the left precentral gyrus, the left middle frontal gyrus, the left middle temporal gyrus, the left caudate, and putamen, as well as the bilateral anterior cingulate gyrus. The reduced FOCA values were mainly found in the right temporal lobe, the bilateral cerebellum posterior lobe, and precuneus. In addition, the FOCA values in the left basal ganglia were linked to durations of epilepsy.

A large range of cognitive dysfunctions and behavioral disturbances in FLE patients, ranging from impairment of executive to the problem of social withdrawal, have been reported in various studies (Braakman et al., 2011, 2012; Exner et al., 2002). Because the frontal lobes play important roles in various functional domains including elemental functions (e.g., motor functions), volitional eye movements, speech and language abilities, motivational behaviors, executive functions, and social competency (Cummings & Miller, 2007), structural and/or functional abnormalities, such as an epileptic focus and/or epileptic activity, within the frontal lobes may account for the cognitive dysfunctions in FLE patients (Braakman et al., 2011; Exner et al., 2002). Using task fMRI, altered functional connections within frontal lobe, as well as connections between the frontal lobe and a wide range of brain regions (including the parietal lobe, temporal lobe, cerebellum, and basal ganglia), have been found in FLE patients (Braakman et al., 2013). Further evidence of resting‐state fMRI studies also showed altered functional connections which were related with the frontal lobes in the FLE patients (Cao et al., 2014; Luo et al., 2014). These findings supported that changes of spontaneous activity in FLE patients may be associated with epileptic activity and cognitive impairment in FLE patients. In our results, increased FOCA values within frontal lobe, including the left precentral gyrus, the left middle frontal gyrus, and bilateral anterior cingulate gyrus, were found in FLE patients. The FOCA measure (Dong et al., 2015), which represents the temporal homogeneity of local adjacent voxels and the regional stability of brain activity states between neighboring time points, is a flexible measure to characterize the local spontaneous brain activity. Therefore, the changes of FOCA in the frontal lobe may reflect functional changes of local spontaneous activity associated with FLE, and to some extent account for potential cognitive impairment in FLE.

Furthermore, in our results, changes of FOCA in left middle temporal gyrus were also found in FLE patients. Using an fMRI memory encoding paradigm, Centeno et al., found that both the frontal and the medial temporal lobe areas were involved in the impairment of memory function in patients with FLE (Centeno et al., 2012). Several studies reported FLE patients to be impaired in functions such as learning and recall, which are supported by temporal lobes (Exner et al., 2002; Helmstaedter, 2010; Nolan et al., 2004). We inferred that changes of FOCA in temporal lobe may be associated with potential impairment of memory function in FLE patients.

It has been suggested that the basal ganglia, which mainly consists of bilateral putamen, pallidum, and caudate, plays an important role in the regulation of epileptic discharges (Norden & Blumenfeld, 2002). Using resting‐state fMRI, compared with controls, abnormal functional integration within the basal ganglia was found in patients with idiopathic generalized epilepsy (IGE) (Luo et al., 2012) and partial epilepsy (Luo et al., 2015). Altered temporal regional homogeneity in the putamen was also found in the patients with rolandic epilepsy (Tang et al., 2014) and mesial temporal lobe epilepsy (Zeng, Pizarro, Nair, La, & Prabhakaran, 2013). Further evidence for the involvement of the basal ganglia in the modulation of epileptic activity has been suggested in several studies of computational evidence in absence seizures (Chen et al., 2014), neuropharmacology (Danober, Deransart, Depaulis, Vergnes, & Marescaux, 1998; Deransart, Vercueil, Marescaux, & Depaulis, 1998), and deep brain stimulation in epilepsy (Loddenkemper et al., 2001). In this study, changes of FOCA values in the left caudate and putamen were found in the FLE patients. Together with these findings, our results supported the view that the basal ganglia may play important roles in the modulation and propagation of epileptic activity in FLE. Of interest, correlations between FOCA values and durations of epilepsy were found in the left basal ganglia in FLE patients. This finding further supported the crucial roles of the basal ganglia in FLE.

Another remarkable finding in our study is the decreased FOCA in the bilateral precuneus. The precuneus and posterior cingulate cortex are crucial nodes of the default model network (DMN) which reflects the baseline brain activities (Buckner, Andrews‐Hanna, & Schacter, 2008; Raichle et al., 2001). In a number of studies, the widespread deactivation in the DMN regions (especially in the precuneus/posterior cingulate cortex), was commonly found in epilepsy patients (Aghakhani et al., 2004; Archer, Abbott, Waites, & Jackson, 2003; Gotman et al., 2005; Hamandi et al., 2006; Li et al., 2009). It has been suggested that epileptic activity may interrupt the resting state and cause the deactivation and suspension of the DMN. Reduced FOCA values in the precuneus suggested reduced consistency of local spontaneous activity in this DMN region. Therefore, we speculated that decreased FOCA in the precuneus of FLE patients may be influenced by the injurious effects of epileptic activity, which may eventually link with the functional impairments of DMN in patients with epilepsy.

Furthermore, decreased FOCA in the bilateral posterior cerebellum was found in FLE patients compared to the healthy controls. Beyond classical involvement of motor coordination, the cerebellum may play roles in multiple functional domains including cognitive, affective, and sensory functions (Diamond, 2000; Schmahmann & Sherman, 1998; Townsend et al., 1999). In the previous studies, changes of local spontaneous activity in the cerebellum were also found in the patients with mesial temporal lobe epilepsy and hippocampal sclerosis (Zeng et al., 2013), juvenile myoclonic epilepsy (Jiang et al., 2016), and absence epilepsy (Yang et al., 2014). In line with these studies, our finding of decreased FOCA in the cerebellum in FLE patients may hint the altered spontaneous neural activity in cerebellum is potentially related to the neurological FLE.

A number of limitations should be acknowledged in this study. First, the sample size (only 19 FLE patients) is modest in this study. Larger sample sizes should be considered in the future to determine our results. Second, because FOCA is a currently new measure, further development (e.g., impacts of number of adjacent voxels on FOCA measure) and application of FOCA will be concerned. Third, the possibility of interictal epileptic discharges may influence the resting‐state consistency of local spontaneous activity in epilepsy patients. Without simultaneously recording EEG during fMRI scans, it is impossible to rule out the effect of confounding epileptic discharges. And, the simultaneous EEG‐fMRI will be considered in the further study to clarify the relationship between FOCA and the interictal epileptic discharges. Fourth, the medication may have influence on our results, despite the fact that these patients had discontinued medication for ~24 hr. Finally, neuropsychological evaluations in FLE patients were not conducted for correlation analysis of behavior with FOCA measure.

## 5 Conclusion

Using a FOCA measure of resting‐state fMRI, the spatiotemporal consistency of local spontaneous activity in FLE patients was investigated in this study. FLE patients demonstrated increased regional consistency in the frontotemporal cortex and basal ganglia. The decreased regional consistency was located in the temporal, cerebellar, and posterior DMN regions. In addition, relationships between FOCA values and duration of epilepsy were found in the basal ganglia. These results indicated that functional changes of local spontaneous activity may be associated with FLE, and may account for the potential cognitive impairment in FLE. The FOCA measure perhaps has potential to provide important insights into understanding the pathophysiological mechanism of FLE.

## Funding Information

The National Nature Science Foundation of China (Grant/Award Numbers: 81271547, 81330032, 81371636, 81471638, 91232725), the 863 Project (Grant/Award Number: 2015AA020505), the Program for Changjiang Scholars and Innovative Research Team (Grant/Award Number: IRT0910), the “111” project of China (Grant/Award Number: B12027), Special‐Funded Program on National Key Scientific Instruments and Equipment Development of China (Grant/Award Number: 2013YQ490859).

## Conflicts of Interest

None of the authors has any conflict of interest to disclose. We confirm that we have read the Journal's position on issues involved in ethical publication and affirm that this report is consistent with those guidelines.
